# A Machine Learning Model for Real-Time Hypoglycemia Risk Prediction in Hospitalized Diabetic Patients: Development and Validation

**DOI:** 10.21203/rs.3.rs-6171081/v1

**Published:** 2025-03-11

**Authors:** Liren Li, Jiayu Chen, Tongshu Guan, Ze Yu, Jinyuan Zhang, Ronghong Ji, Zike Li, Ming Lei, Ping Zheng, Yilei Li, Fei Gao

**Affiliations:** Southern Medical University; Southern Medical University; Southern Medical University; Beijing Medicinovo Technology Co., Ltd; Beijing Medicinovo Technology Co., Ltd; Beijing Medicinovo Technology Co., Ltd; Southern Medical University; Southern Medical University; Southern Medical University; Southern Medical University; Beijing Medicinovo Technology Co., Ltd

**Keywords:** hypoglycemia, Machine-learning models, inpatient, model, Risk prediction

## Abstract

**Background:**

Hypoglycemia is the main obstacle for achieving optimal glucose management in diabetic patients. Despite advances in understanding risk factors, current prediction models for hypoglycemia often rely on static variables and are not optimized for real-time risk assessment in hospitalized patients. This study aims to develop and validate a machine learning (ML)-based prediction model for inpatient hypoglycemia, integrating dynamic clinical data to improve accuracy and clinical utility.

**Methods and Findings:**

We conducted a retrospective study of 37,966 inpatients with diabetes mellitus at Nanfang Hospital, affiliated with Southern Medical University, from January 2021 to December 2022. After applying the inclusion and exclusion criteria, 2,845 patients were included in the final analysis. Data preprocessing focused on analyzing potential predictors, including demographic characteristics, medication use, comorbidities, and laboratory parameters. Through a stepwise forward variable selection method based on XGBoost, we identified 10 optimal predictors. The cohort was randomly split into training and testing sets at an 8:2 ratio. Predictive performance was assessed via the area under the curve (AUC). Ten ML algorithms, including the support vector machine (SVM), CatBoost, XGBoost, random forest, transformer, gradient boosting decision tree (GBDT), TabNet, AdaBoost, light gradient boosting machine (LGBM), and decision tree algorithms, were evaluated. The CatBoost algorithm demonstrated the best performance, achieving an AUC of 0.85, a positive predictive value (PPV) of 0.75, and a negative predictive value (NPV) of 0.89. The model’s decision-making utility was further validated through decision curve analysis and calibration curves, which revealed superior clinical applicability. The key predictors included BMI; insulin use; and laboratory markers such as HbA1c, creatinine, and triglycerides.

**Conclusions:**

Our ML-based predictive model for inpatient hypoglycemia demonstrates robust performance and integrates readily available clinical parameters, offering significant potential for early risk identification and preventive intervention. Future research should focus on multicenter validation and real-time integration into clinical decision support systems to increase generalizability and precision. This study highlights the importance of dynamic data in improving hypoglycemia risk prediction and underscores the potential of ML in advancing diabetes care.

## Introduction

Diabetes mellitus is a complex metabolic disorder characterized by chronic hyperglycemia[1]. Optimal glycemic control remains fundamental in preventing diabetes-related complications, and the management of hypoglycemia has become increasingly critical in the context of individualized treatment paradigms[2]. Recent epidemiological investigations have revealed alarming trends in the prevalence of hypoglycemia across diabetic populations. Large-scale multicenter studies have documented notably high incidence rates: 83% among individuals with type 1 diabetes mellitus (T1DM) and 46.5% among those with type 2 diabetes mellitus (T2DM)[3, 4]. The situation in China is particularly concerning, with reported rates ranging from 32–41% in T2DM patients and reaching 75% in T1DM patients experiencing hypoglycemic episodes[5, 6]. These statistics underscore the magnitude of this clinical challenge and its implications for patient care[2].

Over the past decade, extensive research has been conducted to elucidate the risk factors associated with hypoglycemia. These investigations have focused primarily on three key domains: demographic characteristics, pharmacological interventions, and comorbidities[7, 8]. Demographic factors include age, sex, and body mass index (BMI), whereas medication-related factors include both glucose-lowering agents (particularly insulin and sulfonylureas) and cardiovascular medications such as beta-blockers (BBs), angiotensin-converting enzyme inhibitors (ACEIs), and angiotensin II receptor blockers (ARBs). Comorbidity profiles typically include cardiovascular diseases, cognitive impairment, and hepatic or renal dysfunction, with particular attention given to perioperative conditions[9, 10].

Despite these advances in understanding risk factors, current prediction models exhibit significant limitations. Most existing models rely predominantly on static variables and are optimized for outpatient settings or long-term prognostication. These approaches often fail to incorporate dynamic clinical parameters, such as real-time laboratory results, vital signs, and medication modifications, which are crucial for accurate risk assessment in hospitalized patients. The reliance on preadmission static data alone may inadequately reflect patients’ current physiological status, potentially compromising clinical decision-making[11]. The risk of hypoglycemia in hospitalized patients is dynamic and requires real-time monitoring and assessment. Contemporary hospital information systems possess sophisticated capabilities to capture and integrate dynamic clinical data in real time, including laboratory parameters, vital signs, and medication adjustments. The continuous updating and integration of these data offer the potential to develop more accurate hypoglycemia prediction models. Previous studies have identified several laboratory parameters, including glycated hemoglobin, estimated glomerular filtration rate (eGFR), and albuminuria, as potential predictive markers[12–14]. Furthermore, a study by Ooi reported differences in lipid profile indicators, such as low-density lipoprotein cholesterol (LDL-C) and triglycerides (TGs), as well as renal function markers, such as the eGFR, between patients who experienced hypoglycemia and those who did not[15]. However, these findings require further validation. The emergence of machine learning (ML) has revolutionized healthcare analytics, enabling computers to self-train without explicit programming and to predict outcomes with accuracy[16]. While ML has been increasingly applied to hypoglycemia prediction, there remains substantial scope for improving the precision and clinical utility of the predictive tool[17].

Therefore, this study aims to develop and validate a comprehensive ML-based prediction model for inpatient hypoglycemia. The model integrates multiple data dimensions, including demographic characteristics, medication profiles, comorbidity patterns, and dynamic laboratory parameters, derived from real-world clinical settings. This approach addresses the current limitations in hypoglycemia risk prediction and potentially offers more accurate and clinically applicable prognostic capabilities.

## Methods

### Study Design and Patients

**Figure 1:**
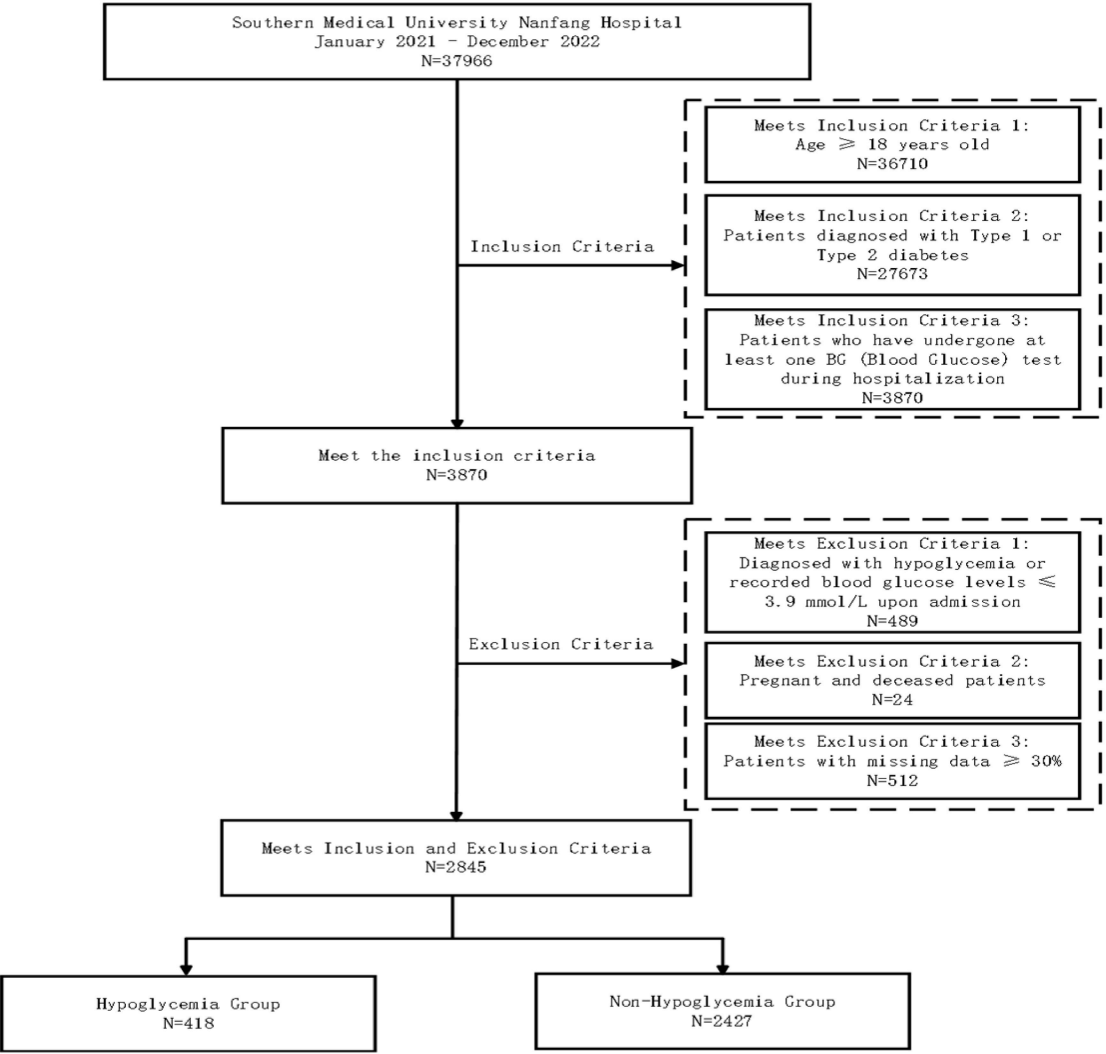
The workflow of patient enrollment. N, number of individuals

The workflow for patient selection is illustrated in Figure 1. This retrospective study included 37,966 inpatients diagnosed with diabetes mellitus at Nanfang Hospital of Southern Medical University between November 1, 2021, and December 31, 2022. All the data were extracted from the hospital’s electronic medical records (EMRs).

The inclusion criteria were as follows: (1) aged ≥18 years; (2) had a confirmed diagnosis of T1DM or T2DM; and (3) had at least one blood glucose (BG) test performed during hospitalization. Patients were excluded if they met any of the following criteria: (1) preadmission hypoglycemia or recorded BG levels ≤3.9 mmol/L; (2) pregnancy or death during hospitalization; or (3) missing data ≥30%. After applying these criteria, 2,845 eligible patients were included in the final analysis.

### Data Preprocessing and Variable Selection

Relevant variables were identified and screened from patient records to determine potential predictors of hypoglycemia. The dataset included four main categories of variables: (1) demographic information (such as age, BMI, sex, smoking and drinking history); (2) medication use (such as insulin, sulfonylureas, β-blockers, and other drugs); (3) comorbidities (such as heart failure, renal insufficiency, and hepatic dysfunction); and (4) laboratory parameters (such as renal function, liver function, and routine blood tests).

To preprocess the data, binary variables were encoded via one-hot encoding, whereas sequential data were processed via sequence coding. Variables with more than 50% missing data or extreme imbalance (positive sample size <0.1% of the total sample size) were excluded. Ultimately, 35 variables were retained for modeling.

**Figure 2:**
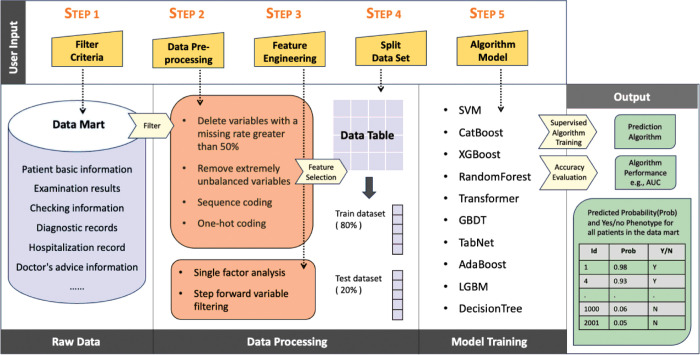
The workflow of data processing and model establishment.

The workflow for data analysis is depicted in Figure 2. Initially, univariate analysis was performed to identify variables significantly associated with hypoglycemia, with a p value threshold of <0.05. A stepwise forward variable selection method based on the XGBoost algorithm was subsequently employed to identify the optimal combination of variables. The model’s performance was evaluated via AUC metric, and the variable combination yielding the highest AUC was selected for further analysis.

### Model development

To ensure robust generalizability, the dataset was randomly split into a training cohort (80% of the data) and a testing cohort (20% of the data). A 10-fold cross-validation was performed on the training dataset to minimize overfitting and ensure the reliability of the predictive model. The specific parameters of the model are shown in Table S1.

As shown in Figure 2, ten ML algorithms were evaluated for their ability to predict hypoglycemia events. These algorithms include support vector machine (SVM), CatBoost, XGBoost, random forest, transformer, gradient boosted decision tree (GBDT), TabNet, AdaBoost, light gradient boosting machine (LGBM), and decision tree. The AUC values of the models generated by these algorithms were compared, and the algorithm with the highest AUC was selected as the final predictive model.

### Statistical analysis

The baseline characteristics of the study population were summarized as frequencies (percentages) for categorical variables and as medians (interquartile ranges, IQRs) for continuous variables, depending on the normality of the data distribution. The Mann–Whitney U test was used to compare continuous variables, whereas categorical variables were analyzed via the chi-square test. Missing values were imputed via the random forest method to ensure data completeness. All the statistical analyses were performed via SPSS software (version 23.0). Model performance metrics, including the area under the curve (AUC), positive predictive value (PPV), and negative predictive value (NPV), were calculated to evaluate the predictive accuracy of the models.

## Results

### Baseline characteristics of the study population

Baseline data were collected from 2,845 hospitalized patients diagnosed with T1DM or T2DM between November 2021 and December 2022. Among these patients, 418 (14.69%) experienced hypoglycemia during their hospital stay. The median age of the cohort was 60 years (IQR: 53–68 years), and 65.83% (n=1,857) were male. The baseline characteristics of the study population are summarized in Table 1(Table 1 is placed at the end of the document, before the References section).

The majority of patients (98.52%, n=2,803) were diagnosed with T2DM, whereas only 1.37% (n=39) had T1DM. In terms of BMI, 41.55% (n=1,182) of the patients had a BMI between 18.5 and 24.0 kg/m^2^, and 32.72% (n=931) had a BMI between 24.0 and 28.0 kg/m^2^. Additionally, 36.73% (n=1,045) of patients reported a history of alcohol consumption, and 43.27% (n=1,231) reported a history of smoking.

The majority of patients (98.52%, n=2,803) were diagnosed with T2DM, whereas only 1.37% (n=39) had T1DM. In terms of BMI, 41.55% (n=1,182) of the patients had a BMI between 18.5 and 24.0 kg/m^2^, and 32.72% (n=931) had a BMI between 24.0 and 28.0 kg/m^2^. Additionally, 36.73% (n=1,045) of patients reported a history of alcohol consumption, and 43.27% (n=1,231) reported a history of smoking.

In terms of medication use, 66.75% (n=1,899) of patients were on insulin therapy, whereas 28.44% (n=809) were prescribed metformin. Other commonly used medications included sulfonylureas (5.83%, n=166), sodium‒glucose cotransporter-2 (SGLT2) inhibitors (17.89%, n=509), and β-blockers (24.82%, n=706). Comorbidities were prevalent, with 7.63% (n=217) of patients having renal insufficiency and 2.92% (n=83) being diagnosed with heart failure. The laboratory parameters, including glycated hemoglobin (HbA1c), creatinine, and albumin levels, are detailed in Table 1.

This is the Table 1 legend. In the “Variable Name” column of the table above, for categorical variables, the format is as follows: “Variable Name, n(%)”, where n represents the frequency of the category, and the value in parentheses indicates the percentage that category represents; for continuous variables, the format is as follows: “Variable Name, median (IQR)”, where median represents the median value, and IQR (interquartile range) represents the lower quartile to upper quartile of the percentage represented by the category.

### Data Preprocessing and Variable Selection

After excluding variables with more than 50% missing data and those with extreme imbalances between positive and negative samples, a total of 27 significant variables were identified through univariate analysis (p<0.05). These variables included demographic factors (such as age class and BMI class), medications (such as insulin, SGLT2 inhibitors, and β-blockers), and laboratory indicators (such as HbA1c, creatinine, triglyceride [TG], albumin, and alanine aminotransferase [ALT]). The results of the univariate analysis are presented in Table 2.

**Figure 3:**
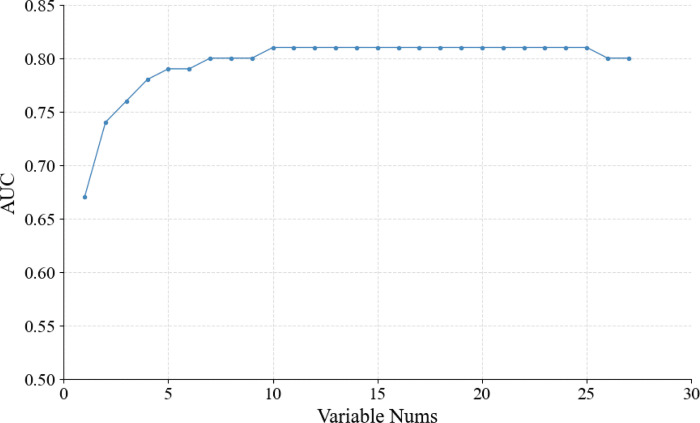
The result of step forward variable selection via XGBoost.

A stepwise forward variable selection method based on the XGBoost algorithm was subsequently applied to identify the optimal combination of variables. As shown in Figure 3, the model achieved the highest AUC value of 0.81 when ten variables were selected. These variables included BMI class; insulin use; and HbA1c, creatinine, urea, TG, albumin, ALT, indirect bilirubin (IDIL), and urine protein (PRO) ***levels*.**

### Model Performance and Interpretation

Ten ML algorithms were evaluated for their ability to predict hypoglycemia events. The predictive performance of these models, assessed via 10-fold cross-validation, is summarized in Table 3. Among the models, the CatBoost algorithm demonstrated the best overall performance, achieving an AUC of 0.85, a PPV of 0.75, and an NPV of 0.89. These metrics outperformed those of the other algorithms, establishing CatBoost as the optimal model for predicting hypoglycemia events.

**Figure 4:**
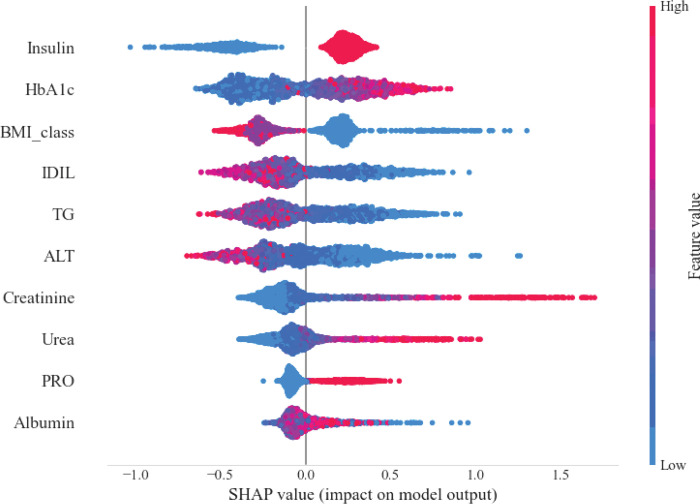
SHAP analysis of the CatBoost model.

Further analysis of the CatBoost model using SHAP (SHapley additive exPlanations) values provided insights into the contribution of individual variables to the model’s predictions. As illustrated in Figure 4, lower BMI values were positively associated with hypoglycemia risk, whereas higher BMI values were negatively associated with hypoglycemia risk. Insulin use was also strongly correlated with an increased likelihood of hypoglycemia. Laboratory markers such as HbA1c, creatinine, and PRO were positively associated with hypoglycemia, whereas ALT, TG, and IDIL were negatively associated with hypoglycemia. Albumin, although significant, demonstrated a more complex relationship with hypoglycemia, as indicated by the SHAP values.

### Decision-Making Utility of the CatBoost Model

The decision-making utility of the CatBoost model was further evaluated via decision curve analysis (DCA) and calibration curve analysis. As shown in Table S1, the DCA demonstrated that the CatBoost model provided the highest net benefit across a range of threshold probabilities, underscoring its clinical applicability. Additionally, the calibration curve analysis revealed a Brier score of 0.088, which was the lowest among all the models, indicating superior accuracy in probability predictions (Table S2).

## Discussion

This comprehensive investigation leveraged electronic health records and advanced ML algorithms to develop a predictive model for inpatient hypoglycemia among patients with diabetes mellitus. Through comparative analysis of ten artificial intelligence techniques, the CatBoost algorithm emerged as the optimal predictive model, achieving remarkable performance metrics (AUC = 0.85, PPV = 0.75) and demonstrating robust predictive accuracy for hypoglycemic events.

Our predictive model identified ten significant variables associated with hypoglycemic events. Notably, BMI and insulin administration emerged as key predictors, which is consistent with established models[18, 19]. The remaining eight variables comprised laboratory parameters, including glycemic control markers (HbA1c), lipid metabolism indicators (TG), hepatic function parameters (IDIL, AST, ALB), and renal function markers (creatinine, UA, PRO). SHAP analysis revealed that insulin utilization was positively correlated with hypoglycemic events, whereas BMI was negatively correlated with hypoglycemic events, which aligns with previous findings[20–23].

Among laboratory parameters, HbA1c has emerged as a crucial predictor of hypoglycemia risk. As illustrated in Fig. 4, elevated HbA1c levels were positively correlated with hypoglycemic events, corroborating the literature[24–26]. Our analysis yielded several novel insights regarding organ function markers. Specifically, hepatic function indicators (IDIL and AST) were inversely correlated with hypoglycemia risk, suggesting potential protective effects of preserved liver function. However, the relationship between albumin levels and hypoglycemia warrants further investigation[27]. Conversely, renal function parameters (creatinine, urea, and PRO) are positively associated with hypoglycemia, indicating that compromised kidney function may constitute a significant risk factor[21]. These findings illuminate the complex interplay between organ system function and hypoglycemia risk, although additional research is necessary for validation.

Despite significant advances in ML-based blood glucose prediction tools over the past decade, this field remains in its early stages[11]. While traditional hypoglycemia risk prediction often emphasizes comorbidities and medications, inpatient glucose prediction necessitates a focus on short-term risk prediction[15, 28]. Given the dynamic nature of hypoglycemia risk, the integration of real-time data with short prediction horizons is crucial for generating clinically actionable insights. Although comorbidity information provides valuable context, it typically reflects admission status rather than current clinical conditions. Conversely, laboratory parameters offer real-time assessment of pathophysiological states, providing the dynamic health information essential for short-term prediction[29].

The CatBoost algorithm, a state-of-the-art method based on the decision tree principle and an advanced iteration of the GBDT algorithm, demonstrated superior predictive capabilities compared with the other nine algorithms[30]. Its performance metrics either match or exceed those of previously published models[11, 27, 31, 32], enabling more precise identification of high-risk individuals and facilitating proactive management strategies.

Several limitations warrant acknowledgment. First, the model’s development utilized single-center, retrospective observational data, potentially limiting generalizability. Second, our analysis did not incorporate lifestyle-related variables, such as dietary patterns and meal timing, which may influence the risk of hypoglycemia.

## Conclusion

We have developed an ML-based predictive model for inpatient hypoglycemia that demonstrates robust performance metrics and utilizes readily available clinical parameters. This model holds potential for integration into clinical decision support systems, enabling early risk identification and preventive intervention. Future directions include system integration for real-time risk prediction in hospitalized diabetic patients, followed by multicenter validation studies to increase model generalizability and precision[33]. Our ultimate objective remains clinical implementation to support evidence-based decisions.

## Figures and Tables

**Table 1.** Baseline characteristics of the study population

	Variable	Median(IQR)|n(%)	Miss rate
Outcome	Hypoglycemia, n(%)		0.00%
	0	2427 (85.31%)	
	1	418 (14.69%)	
Demographics	Age, median (IQR)	60.00 (53.00~68.00)	0.00%
	Age class, n(%)		0.00%
	0(≤65)	1873 (65.83%)	
	1(>65)	972 (34.17%)	
	Gender, n(%)		0.00%
	0(Male)	1857 (65.27%)	
	1(Female)	988 (34.73%)	
	BMI (kg/m^2^), median (IQR)	23.90 (21.70~26.30)	9.24%
	BMI class, n(%)		9.24%
	0(<18.5 kg/m^2^)	113 (3.97%)	
	1(≥18.5 kg/m^2^ and <24.0 kg/m^2^)	1182 (41.55%)	
	2(≥24.0 kg/m^2^ and <28 kg/m^2^)	931 (32.72%)	
	3(≥28 kg/m^2^)	356 (12.51%)	
	Drinking, n(%)		0.00%
	0	1800 (63.27%)	
	1	1045 (36.73%)	
	Smoking, n(%)		0.00%
	0	1614 (56.73%)	
	1	1231 (43.27%)	
	Diabetes, n(%)		0.00%
	0(T1DM)	39 (1.37%)	
	1(T2DM)	2803 (98.52%)	
	2(T1DM+T2DM)	3 (0.11%)	
Medication	Sulfonylureas, n(%)		0.00%
	0	2679 (94.17%)	
	1	166 (5.83%)	
	SGLT2_Inhibitors, n(%)		0.00%
	0	2336 (82.11%)	
	1	509 (17.89%)	
	Glinide, n(%)		0.00%
	0	2775 (97.54%)	
	1	70 (2.46%)	
	DPP4_Inhibitors, n(%)		0.00%
	0	2450 (86.12%)	
	1	395 (13.88%)	
	GLP-1RA, n(%)		0.00%
	0	2724 (95.75%)	
	1	121 (4.25%)	
	AGI, n(%)		0.00%
	0	2391 (84.04%)	
	1	454 (15.96%)	
	TZDs, n(%)		0.00%
	0	2803 (98.52%)	
	1	42 (1.48%)	
	Insulin, n(%)		0.00%
	0	946 (33.25%)	
	1	1899 (66.75%)	
	Metformin, n(%)		0.00%
	0	2036 (71.56%)	
	1	809 (28.44%)	
	Beta-Blockers, n(%)		0.00%
	0	2139 (75.18%)	
	1	706 (24.82%)	
	ACEI/ARB, n(%)		0.00%
	0	1792 (62.99%)	
	1	1053 (37.01%)	
	Levofloxacin, n(%)		0.00%
	0	2646 (93.01%)	
	1	199 (6.99%)	
	Octreotide, n(%)		0.00%
	0	2804 (98.56%)	
	1	41 (1.44%)	
	Warfarin, n(%)		0.00%
	0	2796 (98.28%)	
	1	49 (1.72%)	
	Heparin, n(%)		0.00%
	0	1458 (51.25%)	
	1	1387 (48.75%)	
Diseases	HF, n(%)		0.00%
	0	2762 (97.08%)	
	1	83 (2.92%)	
	Renal insufficiency, n(%)		0.00%
	0	2628 (92.37%)	
	1	217 (7.63%)	
	Hepatic insufficiency, n(%)		0.00%
	0	2814 (98.91%)	
	1	31 (1.09%)	
	Malnutrition, n(%)		0.00%
	0	2832 (99.54%)	
	1	13 (0.46%)	
	Dementia, n(%)		0.00%
	0	2828 (99.40%)	
	1	17 (0.60%)	
	Postoperative, n(%)		0.00%
	0	1361 (47.84%)	
	1	1484 (52.16%)	
Laboratory markers	HbA1c (%), median (IQR)	7.30 (6.50~9.10)	27.45%
	Creatinine (μmol/L), median (IQR)	75.00 (59.00~104.00)	0.42%
	eGFR (ml/min), median (IQR)	88.45 (59.68~102.10)	34.73%
	24hUP (g/24 h), median (IQR)	0.25 (0.11~1.34)	73.39%
	HGB(g/L), median (IQR)	119.00 (98.00~135.00)	0.42%
	Cys-C (mg/L), median (IQR)	1.07 (0.86~1.60)	36.31%
	CRP (mg/L), median (IQR)	7.25 (1.76~36.89)	10.86%
	UA (μmol/L), median (IQR)	304.00 (227.00~390.00)	0.53%
	Urea (mmol/L), median (IQR)	5.90 (4.40~8.30)	0.53%
	LDL-C , median (IQR)	2.57 (1.93~3.32)	28.54%
	HDL-C , median (IQR)	1.00 (0.81~1.20)	28.54%
	TG (mmol/L), median (IQR)	1.54 (1.08~2.26)	28.54%
	TC (mmol/L), median (IQR)	4.21 (3.30~5.24)	28.54%
	ALB (g/L), median (IQR)	37.10 (33.10~40.70)	0.28%
	ALT (U/L), median (IQR)	17.00 (11.00~29.00)	0.28%
	IDIL (μmol/L), median (IQR)	4.70 (3.00~7.20)	0.28%
	ALP (U/L), median (IQR)	89.00 (69.50~120.00)	77.40%
	G (g/L), median (IQR)	26.50 (23.50~29.90)	0.28%
	AST (U/L), median (IQR)	18.00 (14.00~27.00)	0.25%
	DBIL , median (IQR)	3.60 (2.50~5.30)	0.28%
	TBIL (μmol/L), median (IQR)	8.50 (5.80~12.70)	0.28%
	TBA (μmol/L), median (IQR)	6.00 (3.13~18.10)	86.47%
	TP (g/L), median (IQR)	63.90 (59.20~68.60)	0.28%
	PRO, n(%)		12.65%
	0(Negative)	1839 (64.64%)	
	1(Positive)	646 (22.71%)	
	eGFR class, n(%)		34.73%
	0(>120 ml/min)	97 (3.41%)	
	1(≥90 ml/min and ≤120 ml/min)	788 (27.70%)	
	2(≥60 ml/min and <90 ml/min)	502 (17.64%)	
	3(≥30 ml/min and <60 ml/min)	231 (8.12%)	
	4(≥15 ml/min and <30 ml/min)	103 (3.62%)	
	5(<15 ml/min)	136 (4.78%)	
	24hUP class, n(%)		73.39%
	0(<0.15 g/24 h)	274 (9.63%)	
	1(≥0.15 g/24 h and ≤0.5 g/24 h)	198 (6.96%)	
	2(>0.5 g/24 h and ≤4 g/24 h)	196 (6.89%)	
	3(≥4 g/24 h)	89 (3.13%)	
	HGB class, n(%)		0.42%
	0(Male:<120 g/L, Female:<110 g/L)	1273 (44.75%)	
	1(Male:≥120 g/L and ≤160 g/L, Female:≥110 g/L and ≤150 g/L)	1473 (51.78%)	
	2(Male:>160 g/L, Female: >150 g/L)	87 (3.06%)	
	UA class, n(%)		0.53%
	0(Male:<150μmol/L, Female:<89μmol/L)	139 (4.89%)	
	1(Male:≥150μmol/Land ≤416μmol/L, Female:≥89μmol/L and ≤357μmol/L)	2033 (71.46%)	
	2(Male:>416μmol/L, Female:>357μmol/L)	658 (23.13%)	
	Cys-C class, n(%)		36.31%
	0(<0.6 mg/L)	30 (1.05%)	
	1(≥0.6 mg/L and ≤2.5 mg/L)	1535 (53.95%)	
	2(≥2.5 mg/L)	247 (8.68%)	
	CRP class, n(%)		10.86%
	0(≤10 mg/L)	1382 (48.58%)	
	1(>10 mg/L)	1154 (40.56%)	

In the “Variable Name” column of the table above, for categorical variables, the format is as follows: “Variable Name, n(%)”, where n represents the frequency of the category, andthe value in parentheses indicates the percentage that category represents; for continuous variables, theformat is as follows: “Variable Name, median (IQR)”, where median represents the median value, and IQR (interquartile range) represents the lower quartile to upper quartile of the percentage represented by thecategory.

eGFR: estimate glomerular filtration rate; 24hUP: urine protein quantification; HGB: hemoglobin; UA: uricacid; Cys-C: cystatin-C; CRP: C-reactive protein; Diabetes: type of diabetes mellitus; BMI: body mass index; PRO: urine protein; SGLT2: sodium–glucose cotransporter-2 inhibitors; DPP4: dipeptidyl peptidase4 inhibitors; GLP-1: glucagon–like peptide–1 receptor agonists; AGI: alpha–glucosidase inhibitors; TZDs: thiazolidinediones; BBs: β-blockers; ACEI–ARB: angiotensin–converting enzyme inhibitors/angiotensin II receptor blockers; HF: heart failure; HbA1c: hemoglobin A1c; HDL-C: high-density lipoprotein cholesterol; ALB: albumin; IDIL: indirect bilirubin; ALP: alkaline phosphatase; G: globulin; DBIL: direct bilirubin; TBIL: total bilirubin; TBA: total bile acid; TP: total protein.

**Table 2.** Statistical results of univariate analysis.

Variable	P value	Variable	P value
Age class	0.005**	HDL-C	0.989
Gender	0.752	TG	<0.001***
BMI class	<0.001***	TC	<0.001***
Drinking	0.054	Albumin	<0.001***
Smoking	0.926	ALT	<0.001***
SGLT2_Inhibitors	0.029*	IDIL	<0.001***
DPP4_Inhibitors	0.067	Globulin	0.022*
AGI	0.027*	AST	<0.001***
Insulin	<0.001***	DBIL	<0.001***
Metformin	<0.001***	TBIL	<0.001***
Beta_Blockers	<0.001***	Total Protein	<0.001***
ACEI/ARB	0.008**	PRO	<0.001***
Heparin	0.054	eGFR class	<0.001***
Postoperative	0.245	HGB class	<0.001***
HbA1c	<0.001***	UA class	0.750
Creatinine	<0.001***	Cys-C class	<0.001***
Urea	<0.001***	CRP class	0.004**
LDL-C	<0.001***		

*: p<0.05

**: <0.05p<0.01

***: p<0.001

**Table 3.** The predictive performance of the ten models

Algorithm	AUC	PPV	NPV
SVM	0.73	0.65	0.88
CatBoost	0.85	0.75	0.89
XGBoost	0.81	0.71	0.89
RandomForest	0.82	0.71	0.89
Transformer	0.80	0.52	0.90
GBDT	0.79	0.64	0.89
TabNet	0.82	0.42	0.88
AdaBoost	0.74	0.73	0.88
LGBM	0.81	0.72	0.90
DecisionTree	0.72	0.58	0.88

## Data Availability

The datasets used or analysed during the current study are available from the corresponding author on reasonable request.

## Abbreviations

AUC: Area Under the Curve
BBs: Beta-Blockers
BMI: Body Mass Index
BP: Blood Pressure
CGM: Continuous Glucose Monitoring
CRP: C-Reactive Protein
Cys-C: Cystatin-C
DBIL: Direct Bilirubin
DPP4: Dipeptidyl Peptidase 4
eGFR: Estimated Glomerular Filtration Rate
GLP-1: Glucagon-Like Peptide-1
GLP-1RA: Glucagon-Like Peptide-1 Receptor Agonist
HbA1c: Hemoglobin A1c
HDL-C: High-Density Lipoprotein Cholesterol
HF: Heart Failure
IDIL: Indirect Bilirubin
IQR: Interquartile Range
LDL-C: Low-Density Lipoprotein Cholesterol
LGBM: Light Gradient Boosting Machine
ML: Machine Learning
NPV: Negative Predictive Value
PPV: Positive Predictive Value
PRO: Urine Protein
SGLT2: Sodium-Glucose Cotransporter-2
SHAP: SHapley Additive exPlanations
SVM: Support Vector Machine
TBIL: Total Bilirubin
TG: Triglycerides
T1DM: Type 1 Diabetes Mellitus
T2DM: Type 2 Diabetes Mellitus
UA: Uric Acid
